# Limitations of student-driven formative assessment in a clinical clerkship. A randomised controlled trial

**DOI:** 10.1186/1472-6920-8-29

**Published:** 2008-05-11

**Authors:** Edward J Palmer, Peter G Devitt

**Affiliations:** 1 Centre for Learning and Professional Development, University of Adelaide, Adelaide, Australia; 2 Discipline of Surgery, University of Adelaide, Adelaide, Australia

## Abstract

**Background:**

Teachers strive to motivate their students to be self-directed learners. One of the methods used is to provide online formative assessment material. The concept of formative assessment and use of these processes is heavily promoted, despite limited evidence as to their efficacy.

**Methods:**

Fourth year medical students, in their first year of clinical work were divided into four groups. In addition to the usual clinical material, three of the groups were provided with some form of supplementary learning material. For two groups, this was provided as online formative assessment. The amount of time students spent on the supplementary material was measured, their opinion on learning methods was surveyed, and their performance in summative exams at the end of their surgical attachments was measured.

**Results:**

The performance of students was independent of any educational intervention imposed by this study. Despite its ready availability and promotion, student use of the online formative tools was poor.

**Conclusion:**

Formative learning is an ideal not necessarily embraced by students. If formative assessment is to work students need to be encouraged to participate, probably by implementing some form of summative assessment.

## Background

One of the key goals of a medical curriculum is to provide motivation and direction for learning. In the absence of appropriate direction, learning can be an inefficient and time-consuming process, and without suitable goals and guidelines, learners can easily drift away from areas in which they should be focussed. An important role of the teacher is to assist and guide students in their learning; to develop and define appropriate strategies for students and to help them make the most effective use of the time they have available to study. For better or worse a strong stimulus to encourage 'learning' is some form of assessment. Traditionally this has been in the form of summative assessment such as an end-of-course barrier examination. This method focuses students' minds towards a single goal, but tends to foster rote learning with the inevitable "is this going to be in the exam" approach to the choice of material studied. This barrier assessment method governs student decisions on what they will attempt to learn, but is "essentially passive and does not normally have immediate impact on learning" [1]. The impact of summative assessment on the learning process for students should not be underestimated and may have a negative impact on the motivation to learn for some students [2], but "If it's not in the exam, why bother learning it" is an attitude many teachers have encountered.

A preferred stimulus for learning should be some sort of formative assessment process. The concept of formative assessment has been promoted as a means of raising the standards of achievement within the classroom, particularly in primary and secondary education [2]. Formative assessment can be defined as some form of self-assessment by the student, which will provide feedback to both teacher and student. This feedback is then used to modify teaching and learning to meet the student's needs. This strategy has been grasped with enthusiasm, by designers of medical curricula as an apparent means of ensuring deeper learning and understanding. Within the clinical context formative assessment might be used to encourage appropriate professional behaviour, to foster clinical competence and to stimulate acquisition of knowledge and reasoning.

Formative assessment comes in many forms and can vary from informal comments made at the end of a case presentation on a ward round to highly complex and formally structured computer-based learning tools [3,4].

With regard to the latter category, provision of learning materials and study guides are frequently considered suitable tools for formative assessment. However, this is often done without any evidence of their efficacy other than performance in a summative assessment process. In reality, what is required is evidence that the material is used to learn, to stimulate further enquiry and with advantage being taken of feedback. We have undertaken an observational and quantitative study of the use and value of supplementary learning materials provided for formative assessment during a clinical clerkship.

## Methods

Fourth Year clinical students at the University of Adelaide in a nine-week surgical clerkship were enrolled in the study and randomly allocated into one of four groups nominally with equitable distribution according to gender, international status, and academic record. This randomisation was done independent of the study by faculty administrative staff, not because there was any thought that international status or gender would have any effect on results (Table 1).

**Table 1:** Composition of Groups

Group	Percentage International Students	Percentage Male Students	Average Grade end of year 3
A (n = 31)	43%	48%	Credit^1^
B (n = 33)	42%	48%	Credit^1^
C (n = 33)	24%	42%	Credit^1^
D (n = 33)	21%	42%	Credit^1^

^1 ^indicates no significant difference between this and any other group (One-way ANOVA, Bonferoni post hoc test)

Each group was pre- and post-tested for knowledge recall and reasoning using a 50-item multiple-choice examination and a 3-item modified essay question (MEQ) paper. An analysis of the examination material has been described elsewhere [5] and indicated that the Multiple-Choice Questions (MCQs) were of a high quality, tested higher order cognitive skills and had few item writing flaws. The null hypothesis was that exposure to formative assessment material administered in two ways would have no effect on student performance in end-of-attachment assessment. The power of the study was restricted by the number of students available in each group. The authors realised each group would consist of approximately 30 students. They deemed that a good mark for the assessment would be 2/3rds correct ie 20 out of 30 and looked for a 10% difference in outcomes between groups (ie a difference of 2 marks). Previous experience had indicated a standard deviation of approximately 3 was expected. This would provide a power for this study of 0.72 (assuming a 2-tailed type 1 error probability of 0.05)

One group (A) completed the clerkship as standard practice without any additional learning materials other than those recommended to all students within this clerkship (control group). Another group (B) was provided with a series of written case studies. The two other groups (C and D) were given the same case studies as Group B, but in an interactive computer-based format and supplemented with detailed feedback. Group A commenced their clerkship at the beginning of the year. Group D were the last group. At the briefing session at the start of each clerkship students were encouraged to undertake self-directed study throughout the nine weeks and learn about the problems and diseases of the patients with whom they would likely come into contact. There was no mechanism in place to measure if the learning material was accessed in response to encountering patients with these conditions. Where appropriate, either written case studies were provided (Group B) or students were directed to a website [6] containing study material (Groups C and D), and were given instruction on how best to use the provided material. Students were encouraged to use the additional material provided to them and use it to assist them on their ward rounds by discussing the material with colleagues and specialists and using it as a spur to further study. Students were also made aware that there would be a written examination at the end of the clerkship that would contribute towards their overall assessment. All students were provided with references for appropriate texts and websites.

Medici, the software used for the online case studies was developed at the University of Adelaide and has previously been shown to provide learning materials, which students can use to their advantage [4,7]. Medici is an online and CD based program, providing case management problems in a scenario-based context. Students interact with the program either by selecting choices (either single or multiple choice) or by writing answers to questions posed. In both cases, students are provided with instant and detailed feedback on their decisions as well as feedback on the decisions an experienced practitioner might have made. The time spent and work undertaken on the computer-based studies was monitored automatically using this software. There were 12 cases available for student use, approximately 2–3 hours of work in total.

Students in group B had the potential of using the written case notes as a formative learning tool, by examining the material and bringing back questions to experienced specialists on the wards or in tutorials. There was no formal pathway for this to occur, but the students were encouraged to seek feedback from clinicians. A detailed ward report was provided by ward clinicians for every student, and this mechanism was used for providing feedback on areas of strength and weakness to the course coordinator of student teaching. Students in Groups C and D had the additional benefit of receiving automatic and immediate feedback from the Medici program. Students in group C and D differed only in the timing of their attachment.

A questionnaire seeking information on how students used different learning resources during their surgical attachment and their perceptions of the value of these resources was given to each group of students. The resources considered were textbooks, paper based journals, web-based journals, ward activities, lecture, tutorials, non-journal internet resources and interactive teaching aids. The latter category included the provided Medici online cases for groups C and D, but referred to other interactive resource students may have discovered for groups A and B, as these two groups could not access the Medici material. Group B was also asked to consider the written case notes as one of their available resources. The questionnaire was administered at the end of each attachment.

Since differences were present between groups in the pre-test results, the pre-test ability estimates were used as a covariate in the Analysis of Covariance (ANCOVA). In the ANCOVA the post-test ability estimate was used as the dependent variable, the group (A-D) was included as the fixed factor and the pre-test ability estimate was included as a covariate.

The questionnaires were analysed using the Kruskal-Wallis test of ranks. In order to run post hoc contrasts, the data for each significant outcome was ranked and a one-way ANOVA fitted to this ranked data. Post hoc contrasts were performed using Fishers LSD.

## Results

The groups differed slightly in their composition. There were more international students in groups A and B compared to the other two groups. The groups were balanced in gender and in academic ability.

Groups A to D defined four subgroups in the cohort. A significant difference in means was found between pre and post-test results of each group (raw results in Table 2). The performance of the students on the post-test was significantly better than their performance in the pre-test.

**Table 2:** Raw Test Results for Groups A-D.

Group	MCQ Pretest (max score = 46)	MCQ Postest (max score = 46)	MEQ Pretest (max score = 30)	MEQ Posttest (max score = 30)
A (n = 31)	16.0 ± 0.8	26.7 ± 0.6	5.9 ± 0.6	17.4 ± 0.8
B (n = 33)	19.4 ± 0.6	27.3 ± 0.7	5.6 ± 0.5	19.1 ± 0.6
C (n = 33)	21.6 ± 0.7	30.2 ± 0.7	7.6 ± 0.6	18.8 ± 0.7
D (n = 33)	20.8 ± 0.7	28.8 ± 0.8	7.8 ± 0.4	19.7 ± 0.7

Marks are given as score ± standard error

In the ANCOVA the assumption of equality of variance was not violated (Sig value = 0.936 >> 0.05). There was no significant difference in the post-test ability estimates between the different groups after controlling for pre-test ability estimates prior to the interventions. In this case the significance value of 0.232 was greater than 0.05 and it can be concluded that the result is not significant. It can be concluded that there was no overall difference between the 4 groups.

Table 3 shows the reported time spent by students over the whole 9 week attachment using different learning resources. Students reported spending much of their time studying textbooks, working on the wards and attending tutorials (Table 3). Less time was spent using other resources. The value attached to each type of resource by the students, is shown as Table 4. There was no significant difference between groups for the time reported being spent on resources and the value attributed to each resource apart from three instances. Group C reported spending significantly less time on the wards than other groups and Group A valued texts less than other groups. Group C and D valued interactive aids more than group B and Group C also valued them more than Group A.

**Table 3:** Hours spent using various learning resources as reported by students at the end of their attachment.

	Hours spent on learning resources (> 10 hours = 1, 5–10 = 2, 2–5 = 3, 1–2 = 4, <1 = 5)								
Group	Texts	Journal (Paper)	Journal (Web)	Ward^1^	Lectures	Tutorials	Interactive aids	Internet (non-journal)	Other

A	1 [1-1]	4 [2–5]	3 [2–4]	1 [1-1]	2 [1–3]	1.5 [1–2]	4 [3–5]	4 [2–5]	3 [2–5]
B	1 [1-1]	4 [3–5]	2 [1–3]	1 [1-1]	2 [1–4]	2 [1–2]	5 [4–5]	3 [2–5]	4.5 [1–5]
C	1 [1-1]	4 [3–5]	3 [2–4]	1^2 ^[1–2]	2 [2–4]	2 [1–2]	4 [3–5]	4 [3–5]	4 [3–5]
D	1 [1-1]	4 [4–5]	3 [2–4]	1 [1-1]	2 [2–3]	1.5 [1–2]	5 [3–5]	3 [2–4]	5 [5-5]
All groups	1 [1-1]	4 [3–5]	3 [2–4]	1 [1-1]	2 [1–3]	2 [1–2]	4 [3–5]	3 [2–5]	5 [3–5]

1 = more than 10 hours, 2 = 5–10 hours, 3 = 2 to 5 hours, 4 = 1–2 hours, 5 = less than 1 hour Results are expressed as median [interquartile range]

1 Indicates significant difference between groups (Kruskal-Wallis test, p < 0.05)

2 Significant difference between this group and all other groups (One way ANOVA on Ranks, Fishers LSD post hoc test, p < 0.05)

**Table 4:** Perceived value of learning resources as reported by students at the end of their attachment.

	Perceived value of resources (very valuable = 1, valuable = 2, little value = 3, no value = 4)								
Group	Texts^1^	Journal (Paper)	Journal (Web)	Ward	Lectures	Tutorials	Interactive aids^1^	Internet (non-journal)	Other

A	1^2^ [1-1]	2 [2–3]	2 [1–2]	2 [1–2]	2 [2-2]	1 [1-1]	2 [2–3]	3 [2–3]	2 [2–4]
B	1 [1-1]	2 [2–3]	1.5 [1–2]	2 [1–2]	2 [2-2]	1 [1–2]	3 [2–3]	3 [2–3]	2.5 [1–3]
C	1[1-1]	2 [2–3]	2 [1–2]	2 [2-2]	2 [1–2]	1 [1-1]	2^3,4 ^[1–3]	2 [2–3]	3 [2–3]
D	1 [1-1]	2 [2–3]	2 [1–2]	2 [1–3]	2 [1–2]	1 [1–2]	2^3 ^[2–3]	2 [–-3]	3 [3–4]
All groups	1 [1-1]	2 [2–3]	2 [1–2]	2 [1–2]	2 [1–2]	1 [1–2]	2 [2–3]	2 [2–3]	3 [2–3]

1 = very valuable, 2 = valuable, 3 = little value, 4 = no value Results are expressed as median [interquartile range]

1 Significant difference between groups (Kruskal-Wallis test, p < 0.05)

2 Significant difference between this group and all other groups (One way ANOVA on Ranks, Fishers LSD post hoc test, p < 0.05)

3 Significant difference between this group and Group B. (One way ANOVA on Ranks, Fishers LSD post hoc test, p < 0.05)

4 Significant difference between this group and Group A. (One way ANOVA on Ranks, Fishers LSD post hoc test, p < 0.05)

The results also show that students attached most value to textbooks and tutorials, but indicated that they felt most resources were valuable in their own right. Students in both groups A and B reported spending some time using interactive material and also rated its value. The source of this material is unknown. It was not the online material made available to groups C and D, as it had not yet been developed.

For the group provided with handouts (group B) the students spent a median time of 3 (ie about 2 hours in total over the nine weeks) (interquartile range 3–4) on the material and gave it a value of 2 (interquartile range 1–2) (ie valuable).

The use of the online formative assessment tool was monitored for each group over the period from the beginning of their attachment to the end of the academic year. Groups A and B were given access after their attachments (corresponding to the time the material was ready for use) and made little use of the material. Groups C and D were encouraged to use the resource and although greater use of it was made, less than 30% of group C and only 20% of group D accessed the material (Table 5). Where the material was used, students often revisited the available cases and spent over an hour studying the case material.

**Table 5:** Student usage of the Medici interactive online resource from beginning of attachment to the end of the academic year.

Group	No students attempting cases (total no. students)	Median number of cases attempted	Median number of screens (approx 10 screens per case)	Median time spent (minutes)
A	4 (34)	2 [1.5–5]	40 [12.5–63]	28 [9–30]
B	2 (33)	3.5 [3–4]	37 [14–60]	25 [9–42]
C	11 (28)	11 [6–12]	129 [59–240]	90 [20–143]
D	6 (30)	7 [2–10.5]	82.5 [17–152]	53 [25–140]

Results are expressed as median [interquartile range]

Thirty percent of all monitored activity occurred after the surgical attachment and was presumably used for exam preparation at the end of the year.

## Discussion

We have shown that while students improved their knowledge and understanding during the nine-week surgical attachment, any advice and help they were offered with regard to self-directed study and formative assessment did not appear to produce any variation in their improvement in cognitive skills or change in study habits. Despite clear guidance at the beginning of the course on the goals of the attachment and how students might help themselves with their learning, little attention appears to have been paid to this advice. The intention of the course was that students should study as they went along and – for the appropriate groups – were given guidelines of when and what to study. The idea of the written and computer-based material was that students would be able to see what standards were expected and gain feedback on their individual performance. Teachers would also benefit from examining the performance of students, especially in the online environment, where it would be possible to examine misconceptions and act on them or at least provide prompt feedback. This fits with the generally accepted concept of 'formative assessment [1,9,10].

Although groups A and B contained more international students than the other two groups, the groups were academically equivalent. The authors do not believe that this affects the results of the study, but is nonetheless a potential confounding factor. The survey results from group C showed small differences in attitude and reported behaviour compared to other groups, but these results were not completely repeated in group D, thus making it difficult to generalise. Although Groups C and D did make use of the online material during their attachments, the amount of use was disappointing. This apparent lack of interest would be unlikely to impress Departments and Faculties that devote substantial time, effort and money into the production and presentation of materials for online formative assessment. In the case of Medici, this lack of interest was despite the fact that the software program has been promulgated within the Faculty and has been reported in the international literature as to its worth as a source of adjunct learning [7,8]. Low compliance with online educational modules has been reported [11,12] but the reason behind this is not apparent, although technical issues and lack of time have been raised as issues [11]. Whilst it could be argued that the aim of formative assessment is not so much to raise the standards of attainment [2] but to foster the spirit of learning, it appears clear that neither goals were achieved in this case by the formative material provided. On the other hand, once students did begin making use of the formative online resource, they often made frequent use of it. It may be that the biggest issue involved in encouraging students to use this type of formative assessment is by discovering a method to encourage the students to begin using it.

The key motivating factors associated with assessments are the perceived relevance of the assessment, the content of the assessment, the enthusiasm of lecturers and group influences [13]. The first two items are related directly to strategic considerations: 'can I learn what I need to pass or be good at what I want to do'. The last two items are external influences and can be controlled to some extent but may be difficult to implement in environments where the teacher is distant from the student, such as the online realm or where the teachers are busy clinicians and may not have the time to enthuse students or build a supportive group structure. This appears to have been reflected in our study, where students apparently placed value on the material provided for them but in reality, did not make much use of it. When they did use the formative material a large proportion of it was in immediate preparation for a summative examination.

As well as the methods used by the teacher to facilitate learning, there are other factors affecting student motivation, including student goals and interests, creativity and the willingness to learn [14]. Thus extrinsic motivation is more difficult to apply as some students are easily distracted, tend to take short-cuts or get jaded and lose interest as they go further along a course [15]. A substantial proportion of students have part-time employment and they may focus on this rather than clinical activities [15]. If students are not motivated to work on clinical activities, what would motivate them to work on an online formative assessment tool?

This study was undertaken on the premise that sufficient motivation would be provided to the students by stressing the importance of the formative content and linking the formative assessment material to the learning objectives of the course. Regular feedback, likely to improve student's work ethic [16], was provided to the students by the formative tool, and added incentive was provided, by informing students that this content was examinable.

The nature of an assessment can be a key indicator of the effort students will put into assessment tasks [17], but students are capable of manipulating their study time to focus on examinations at the expense of understanding subject matter [16]. Deep learning is one of the goals of any course and "if students perceive a need to understand the material in order to successfully negotiate the assessment task, they will engage in deep learning [18]."

The good teacher will appreciate that an important goal is learning itself for self-improvement. This is particularly important in medicine where much professional interest is focussed on continuing medical education and credentialing, but these values are often difficult to appreciate at student level, when the barrier of final examinations looms large. This type of behaviour has been observed in medical education, where students learn those elements of the curriculum that are known to be directly assessed and are more concerned about grades or passing an examination than about using assessments as a learning experience [19], i.e. 'know what they need to know' for the examination rather than to improve their overall competence. It is this high stakes process that is often considered to have a negative impact on formative assessment [14].

A number of criteria have been defined to guide assessment practices [16]. Although the written cases notes had the potential to meet these criteria, the Medici online program dealt with many of these explicitly. The program content provided to students in our study met many of these suggested criteria, including providing sufficient tasks to utilise available study time, engaging the students in an appropriate activity and providing instant, relevant and complete feedback in sufficient detail, which was focussed on student learning. One of the criteria relies on communicating clear and high expectations to the students. Although it was believed that this was done via assessment notes and verbal communication, it may be that the students failed to grasp this fact, or that there was a failure to re-enforce this message.

## Conclusion

If academic staff are going to prepare formative learning material for students, there must be some indication that the effort would be worthwhile. It is clear from this study that the strategies and materials provided to students failed to motivate them and also to make any meaningful difference to their ability to pass a standard summative assessment, thus making the process a failure from the point of view of both parties. Strategies need to be put into place, which will engage and motivate the students to use resources which teachers believe will enhance their learning.

## Competing interests

The authors declare that they have no competing interests.

## Contribution

EJP, PGD contributed to study design, data collection and interpretation. They both contributed to all versions of the manuscript.

## Pre-publication history

The pre-publication history for this paper can be accessed here:


